# Political events and mood among young physicians: a prospective cohort study

**DOI:** 10.1136/bmj.l6322

**Published:** 2019-12-09

**Authors:** Elena Frank, Brahmajee K Nallamothu, Zhuo Zhao, Srijan Sen

**Affiliations:** 1 Molecular and Behavioral Neuroscience Institute, University of Michigan, Ann Arbor, MI, USA; 2 Michigan Integrated Center for Health Analytics and Medical Prediction and Division of Cardiovascular Medicine, Department of Internal Medicine, University of Michigan, Ann Arbor, MI, USA; 3 Molecular and Behavioral Neuroscience Institute, University of Michigan, Ann Arbor, MI, USA; 4 Department of Psychiatry, University of Michigan, Ann Arbor, MI, USA

## Abstract

**Objective:**

To study the effects of recent political events on mood among young physicians.

**Design:**

Prospective cohort study.

**Setting:**

United States medical centres.

**Participants:**

2345 medical interns provided longitudinal mood data as part of the Intern Health Study between 2016 and 2018.

**Main outcome measures:**

Mean mood score during the week following influential political and non-political events as compared with mean mood during the preceding four week control period.

**Results:**

We identified nine political events and eight non-political events for analysis. With the start of internship duties in July, the mean decline in mood for interns was −0.30 (95% confidence interval −0.33 to −0.27, t=−17.45, P<0.001). The decline in mood was of similar magnitude following the 2016 presidential election (mean mood change −0.32, 95% confidence interval −0.45 to −0.19, t=−4.73, P<0.001) and subsequent inauguration (mean mood change −0.25, 95% confidence interval −0.37 to −0.12, t=−3.93, P<0.001). Further, compared with men, women reported greater mood declines after both the 2016 election (mean gender difference 0.31, 95% confidence interval 0.05 to 0.58, t=2.33, P=0.02) and the inauguration (mean gender difference 0.25, 95% confidence interval 0.01 to 0.49, t=2.05, P=0.04). Overall, there were statistically significant changes in mood following 66.7% (6/9) of political events assessed. In contrast, none of the non-political events included in the analysis were statistically significantly associated with a change in mood.

**Conclusions:**

Macro level factors such as politics may be correlated with the mood of young doctors. This finding signals the need for further evaluation of the consequences of increasing entanglement between politics and medicine moving forward for young physicians and their patients.

## Introduction

Over the past decade, growing and much needed attention has been paid to high rates of depression experienced by training physicians. Several systemic factors, including heavy workloads, medical errors, and sleep deprivation have been implicated as factors compromising the wellbeing of young doctors.^1^ ^2^ ^3^ Less studied is the impact of exogenous factors such as dramatic societal events—including politics—on the mental health of training physicians. On one hand, the busy day-to-day life of training physicians may make them impervious to such factors. Alternatively, high baseline levels of stress at work may lead to less resilience and large swings in emotions during turbulent events.

In the current era, the 2016 US presidential election stands out as a singular political event. Although doctors have traditionally sought to keep politics and medicine separate, changing demographics in medicine and growing debate around issues such as healthcare reform and women’s reproductive health have made intersections between medicine and politics increasingly unavoidable.^4^ ^5^ ^6^ ^7^ Beliefs about politicised health issues can influence physicians’ treatment decisions, and increasing levels of political engagement among physicians may have both personal and public health consequences.^8^ Further investigation of the extent to which the current generation of young physicians may be affected by politics could be useful to better understand implications for physician wellbeing and patient care.

Using long term data on mood from the Intern Health Study, we sought to examine the effect of political events in the contemporary era on young physicians.^9^ We used Google Trends, a tool increasingly employed in health research for gauging population behaviour, to identify periods of peak national awareness of key societal events related to politics.^10^ In the wake of the 2016 presidential election, we hypothesised that interns would experience a greater change in mood following political events compared with other major events that were non-political.

## Methods

### Participants

The Intern Health Study is a prospective cohort study assessing stress and depression during the first year of residency training in the US.^1^ In total, 615, 537, and 2129 incoming interns were enrolled in the daily mood arm of the study during the 2016-17, 2017-18, and 2018-19 academic years, respectively, of which 2345 were included in the current analysis. Participants represented 12 specialties at more than 300 residency institutions across the US (Northeast: 25.6%, Midwest: 31.9%, South: 28.0%, West: 14.6%) (table 1) and received $50 (2016 and 2017 cohorts) or $125 (2018 cohort) in compensation. The study was approved by the University of Michigan Institutional Review Board and we obtained informed consent from all study participants.

**Table 1:** Sample demographic characteristics

Characteristics	Number of subjects (%)
Age, mean (standard deviation), year	28 (2.62)
**Gender**	
Female	1301 (55.48)
Male	1044 (44.52)
**Ethnicity**	
White	1411 (60.27)
Asian	478 (20.42)
African American	105 (4.49)
Latino	92 (3.93)
Arab/Middle Eastern	42 (1.79)
Native American	1 (0.04)
Multi racial	207 (8.84)
Other	9 (0.21)
**Specialty**	
Internal medicine	528 (22.54)
Paediatrics	327 (13.96)
General surgery	214 (9.13)
Emergency medicine	211 (9.01)
Family medicine	190 (8.11)
Obstetrics/gynaecology	152 (6.49)
Psychiatry	127 (5.42)
Anaesthesiology	119 (5.08)
Internal medicine/paediatrics	64 (2.73)
Neurology	45 (1.92)
Otolaryngology	41 (1.75)
Transitional	72 (3.07)
Other	255 (10.80)
**Marital status**	
Single or separated	1445 (61.62)
Married or engaged	900 (38.38)

### Data collection

To understand the effects of politics on the mental health of young physicians, we assessed how the most salient societal events that occurred during our study period changed the daily mood of interns. We stratified these by political and non-political events. Before the start of the internship, subjects completed an initial survey where they provided demographic information, including gender. Throughout the intern year, subjects responded daily to the following validated one-question measure of mood valence via the Intern Health iPhone app: “On a scale of 1-10 how was your mood today?”^11^ ^12^ Subjects were prompted through an app notification to submit a mood score daily at 8 pm.

We identified political and non-political events that had the greatest impacts since the 2016 presidential election based on a *History Channel* summary of notable 2017 and 2018 events.^13^ ^14^ Events categorised as “Politics” were selected as the political events in our analysis. However, for the purposes of this study we included only domestic events in the United States. In addition to the 2016 presidential election, we identified eight political events for inclusion in the analysis (box 1).

Box 1 **Political events**

**Presidential election**

Donald Trump is elected president in the US national election

**Presidential inauguration**

Donald Trump is inaugurated as the 45th president of the US

**Muslim travel ban**

A US presidential executive order is signed banning nationals from seven Muslim majority countries and refugees from Syria and other nations from visiting the US

**Failure to repeal the Affordable Care Act**

The US Senate rejects the third of a sequence of proposals to repeal and replace the Affordable Care Act

**Executive order to prevent the separation of immigrant families at the US-Mexico border**

A US presidential executive order intended to keep migrant families together is signed soon after the release of a government report of the separation of almost 2000 immigrant children from their families as part of the “zero tolerance” policy at the US-Mexico border resulted in a national outcry

**Kavanaugh Supreme Court confirmation**

Judge Brett Kavanaugh is nominated to replace Justice Anthony Kennedy on the US Supreme Court. Kavanaugh is confirmed despite controversy surrounding allegations of sexual assault

**Migrant caravan**

The US presidential administration deploys active duty military troops to the US-Mexico border to meet a large migrant caravan from South America

**Midterm elections**

US midterm elections were held and resulted in the Democratic party gaining a majority in the House of Representatives. The Republican party retained its majority in the Senate

**Failure to pass budget including US-Mexico border wall funding**

A budget bill requesting $5 billion in federal spending on a US-Mexico border wall fails to pass in the US Senate

We considered all other events, categorised as either “Culture” or “Health, Science, and Environment,” for inclusion as non-political events (box 2). A few events listed under “non-political” could be considered political in nature (eg, women’s march on Washington, National Football League anthem protests); we excluded these after independent and consensus assessment by two of the authors, before analyses were performed.

Box 2 **Non-political events**

**Super Bowl LI**

The New England Patriots stage the largest comeback in Super Bowl history to defeat the Atlanta Falcons

**Solar eclipse**

A total solar eclipse was visible across the entire continental US, the first since 1918

**Hurricane Irma**

In the wake of devastation caused by Hurricane Harvey, Irma makes landfall, becoming the strongest hurricane to hit the US since Katrina in 2005

**Las Vegas shooting**

A gunman kills 58 and injures more than 500 people in a mass shooting at a Las Vegas music festival

**Shooting at Florida high school**

A mass shooting at a high school in Parkland, Florida results in the death of 17 students and staff, and the injury of 17 others

**Royal wedding of Prince Harry and Meghan Markle**

American actress Meghan Markle weds Prince Harry, a member of the British royal family

**Hurricane Florence**

Hurricane Florence causes more than 50 deaths in Virginia and the Carolinas

**California wildfires**

Destructive wildfires ravage California. The Camp Fire results in more than 90 fatalities, becoming the deadliest fire in state history

For each event, we queried Google Trends (accessed July 23, 2019) to determine the date of peak public interest (value of 100) within the US. We determined search terms by author consensus based on keywords used in the *History Channel* event summary, and in some cases we used multiple search terms (supplementary file, table 1). We followed the Checklist for Documentation of Google Trends.^10^

### Statistical analysis

We used paired t-tests to compare the mean mood for the week following an event (as defined by peak interest on Google Trends) with the mean mood during the four weeks preceding the event. For events associated with a statistically significant mood change, we first determined the percentage change in mood for men and women and then used a two-sample t-test to determine whether there was a statistically significant gender difference in mood change. We also conducted a sensitivity analysis where we modeled the change in mood score with the event while including the baseline mood score before the event as a covariate. In addition, to explore for geographic variability in our results, we performed a series of one-way analyses of variance to assess for mood change differences in response to events between the four US census regions. Finally, to globally assess whether there was a systematic difference between political and non-political events on their effects on mood, we ran a general linear regression with the absolute value of mood change score for each of the 17 events as the outcome with the political/non-political nature of each included as a covariate. All analyses were performed using SAS version 9.4. P values less than 0.05 were considered statistically significant.

## Results

In addition to the 2016 presidential election, we identified eight political events and eight non-political events to study (table 2). Of the enrolled interns, 71.5% (2345/3281) entered a daily mood score during at least one included event period and four weeks preceding that event and were included in the analysis (table 1 gives participant information). Responders were slightly older than non-responders (27.6 years versus 27.3 years; P=0.001) but the groups were not statistically significantly different with respect to gender or change in depression rates with internship.

**Table 2:** Change in mean mood score from the four week period preceding the week of the date of peak interest (as determined by Google Trends) for political and non-political events

Political events	Date range	Mood change (95% confidence interval)	t value	P value
Presidential election	9-15 November 2016	−0.32 (−0.45 to −0.19)	−4.73	<0.001
Presidential inauguration	20-26 January 2017	−0.25 (−0.37 to −0.12)	−3.93	<0.001
Muslim ban	29 January-4 February 2017	−0.21 (−0.34 to −0.07)	−3.07	0.002
Failure to repeal Affordable Care Act	28 July-3 August 2017	−0.07 (−0.15 to 0.01)	−1.67	0.10
Executive order to prevent border separations	20-26 June 2018	0.16 (0.01 to 0.30)	2.10	0.04
Kavanaugh confirmation	28 September- 4 October 2018	−0.06 (−0.12 to −0.01)	−2.35	0.02
Migrant caravan	23-29 October 2018	−0.03 (−0.09 to 0.03)	−1.04	0.30
Midterm elections	7-13 November 2018	−0.03 (−0.08 to 0.03)	−0.95	0.34
Failure to pass border wall funding	21-27 December 2018	0.17 (0.11 to 0.23)	5.28	<0.001
				
Non-political events	Date range	Mood change (95% confidence interval)	t value	P value
Super Bowl LI	6-12 February 2017	0.06 (−0.07 to 0.18)	0.92	0.36
Solar eclipse	21-27 August 2017	0.02 (−0.08 to 0.12)	0.42	0.67
Hurricane Irma	6-12 September 2017	−0.09 (−0.18 to 0.01)	−1.79	0.07
Las Vegas shooting	2-8 October 2017	−0.08 (−0.18 to 0.02)	−1.51	0.13
Florida high school shooting	15-21 February 2018	−0.09 (−0.22 to 0.05)	−1.29	0.20
Royal wedding	19-25 May 2018	−0.04 (−0.17 to 0.09)	−0.60	0.55
Hurricane Florence	13-19 September 2018	−0.06 (−0.12 to 0.01)	−1.70	0.09
California wildfires	15-21 November 2018	−0.04 (−0.10 to 0.02)	−1.35	0.18

Overall, responding interns reported notable changes in mood following six of the nine political events. The largest decline in mood was observed after the 2016 presidential election (mean mood change −0.32, 95% confidence interval −0.45 to −0.19, t=−4.73, P<0.001), with statistically significant declines in mood also following the January 2017 inauguration (mean mood change −0.25, 95% confidence interval −0.37 to −0.12, t=-3.93, P=0.001), the ban on travel from Muslim majority countries (mean mood change −0.21, 95% confidence interval −0.34 to −0.07, t=−3.07, P=0.002), and Supreme Court confirmation hearings in September 2018 (mean mood change −0.06, 95% confidence interval −0.12 to −0.01, t=−2.35, P=0.02) (table 2). We identified statistically significant increases in mood following the signing of a US presidential executive order designed to keep migrant families together at the US Mexico border (mean mood change 0.16, 95% confidence interval 0.01 to 0.30, t=2.10, P=0.04) and the failure to pass a federal spending bill that included funding for a border wall (mean mood change 0.17, 95% confidence interval 0.11 to 0.23, t=5.28, P<0.001). As a reference and to place these changes in context, the change in mood score associated with the start of internship duties in July was −0.30 (95% confidence interval −0.33 to −0.27, t=−17.45, P<0.001) for our overall sample. Among those subjects who developed depression during internship, the change in mood score was −0.81 (95% confidence interval −0.88 to −0.75, t=−23.81, P<0.001). These findings suggest some of the changes reported above were comparable to declines in mood seen during the start of internship but less than the declines seen in those who developed depression.

Not all political events were associated with statistically significant changes in mood score. No difference in mood was observed with the failure to repeal the Affordable Care Act in the US Senate (mean mood change −0.07, 95% confidence interval −0.15 to 0.01, t=−1.67, P=0.10), the deployment of troops to the Mexico border to meet a large migrant caravan (mean mood change −0.03, 95% confidence interval −0.09 to 0.03, t=−1.04, P=0.30), or the 2018 midterm elections (mean mood change −0.03, 95% confidence interval −0.08 to 0.03, t=−0.95, P=0.34). In contrast to the political events, none of the non-political events included in the analysis were statistically significantly associated with a change in mood. In a global analysis across all 17 events, we found that the absolute value of mood change after political events was statistically significantly greater than after non-political events (mean mood change difference 0.09, 95% confidence interval 0.16 to 0.005, F=5.09, P=0.04). In our sensitivity analyses, we confirmed the same six political events were statistically significantly associated with a change in mean mood score after the event when including the baseline mood before the event as a covariate. In contrast, there were no statistically significant time effects for the three remaining political events or any of the non-political events (findings not shown but available from authors).

Some gender differences existed in our findings. Women experienced a greater decline in mood with the US presidential election compared with men (mean gender difference 0.31, 95% confidence interval 0.05 to 0.58, t=2.33, P=0.02), and a greater decline in mood in response to the inauguration (mean gender difference 0.25, 95% confidence interval 0.01 to 0.49, t=2.05, P=0.04). In contrast, men experienced a greater mood increase when the Senate failed to pass federal funding to build a border wall (mean gender difference 0.13, 95% confidence interval 0.01 to 0.26, t=2.08, P=0.04).

Across the four geographic regions of the US, we noted no statistically significant difference in mood score change for 15 of 17 events. For the 2016 US presidential election (F=3.5, P=0.02) and January 2017 inauguration (F=3.2, P=0.03), the South region had smaller declines in mood compared to the Northeast, Midwest, and West.

## Discussion

Our findings describe the susceptibility of young US physicians’ moods to major political events during arguably one of the hardest periods of their work lives: intern year. Although numerous factors related to their daily work schedule have been described extensively, the impact of exogenous factors such as those examined here has not been previously reported.

We found that the decline in mood with the 2016 US presidential election was greater than the decline with the start of internship—a transition associated with a considerable increase in stress and a fivefold increase in depression.^1^ ^15^ This suggests that, even with the high demands and time constraints of internship, young US physicians were engaged with broader sociopolitical events. By comparison, we found that non-political events did not meaningfully affect mood in these young physicians in aggregate.

The directionality of these findings is consistent with evidence that young voters and voters with postgraduate education tend to identify as liberal leaning, and supports previous work showing a strong left shift in political affiliation among physicians over the past 25 years.^6^ ^16^ With Republican campaign pledges to repeal the Affordable Care Act and restrict women’s access to reproductive health services domestically and abroad, these young physicians may have been especially concerned about the healthcare consequences of a Republican presidency.

We also found that women were particularly affected by the election results. Following the presidential election and subsequent inauguration, women experienced mood declines that were more than double that of their male counterparts. This finding suggests that the political discourse surrounding issues of gender and sexism throughout the presidential campaign may have disproportionately affected women. The gender difference may have also reflected a greater disappointment among women interns that the US did not elect its first female president. Female interns in our sample may have thus experienced the election outcome on both political and personal levels.

Political events continued to correlate with interns’ mood after the January 2017 presidential inauguration. Events with outcomes that aligned with conservative political ideologies, such as the Muslim travel ban and Brett Kavanaugh’s confirmation to the US Supreme Court, were associated with a mood decrease. In contrast, events with outcomes in line with liberal political ideologies were followed by a mood increase, including the signing of a US presidential executive order to keep migrant families together at the US-Mexico border and following the Senate’s failure to pass funding for a border wall. These findings further support existing evidence that young physicians may increasingly identify as liberal, particularly around factors such as gender, ethnicity, and nationality.^6^

For most political and non-political events, there was no statistically significant difference in mood change across the four primary US geographic regions. The exceptions to this trend were the presidential election and inauguration, with interns in the South experiencing smaller declines than interns in other regions. A higher proportion of the general population voted Republican in the 2016 presidential election in the South (51.8%) than in the Northeast (40.5%), Midwest (49.2%), or West (38.0%), suggesting that geographic differences in response to the highly partisan election and inauguration events among interns may reflect regional variation in political affiliation.^17^

### Implications

What possible mechanisms could underlie our overall findings? In a time defined by the 24 hour news cycle and instantaneous social media updates, exposure to political news is not only unavoidable, but constant. Acute media exposure to severe violence or disasters, such as the September 11 attacks on the World Trade Center, has been shown to negatively affect mental and physical health and even result in symptoms akin to post-traumatic stress disorder (PTSD).^18^ Our findings suggest that, in recent years, repeated long term exposure to emotionally arousing news can also have psychological implications. While not as severe as PTSD, these emotional ups and downs may still add to the mental burden of young US physicians, who are already under high levels of stress and at increased risk for mental health issues.^1^ ^2^ ^3^

Previous research indicates that events like national elections can be experienced as stressful life events with psychological and biological consequences.^19^ ^20^ ^21^ The 2016 US presidential election has been linked to increases in psychological distress, and short term mood changes following the election associated with more sustained physiological stress responses among young adults.^22^ ^23^ Studies have also shown an increase in psychological concerns and preterm births among Latina women following the 2016 election.^24^ ^25^ Along with our data, this suggests that large scale political events can influence factors relevant to mental and physical health, particularly for those with specific concerns about how the events may affect their lives. For physicians, however, this may extend beyond the personal implications. Shifts in mood among specific groups following sociopolitical events could also have professional consequences as physicians regularly interact with diverse populations.^26^ With our finding that political events are associated with changes in mood among young physicians in the US, future studies should examine whether similar dynamics are playing out for young physicians in other countries. In the UK, for example, there may also be emotional consequences for physicians increasingly concerned about the ramifications of Brexit for themselves and their patients.^27^

Leading medical organisations have emphasised the need for separation between medicine and politics throughout much of the 20th century.^28^ ^29^ Data from the present study suggests that maintaining this separation may be a challenge for the current generation of young physicians who appear to experience mood variations with major sociopolitical events. As residency is a period already characterised by high stress and risk for depression, emotional instability surrounding politics could have personal health implications. At the same time, as physicians’ treatment decisions can be influenced by feelings about politics, this could also lead to consequences for patient care.

### Limitations

Our study has several limitations. Because our sample consisted of first year intern physicians, results may not be generalisable to all doctors or to other young, politically liberal populations. While we focused on the objectively most salient political and non-political events during the study period, other individual or societal level events affecting mood may have occurred during our study periods and confounded our results. Further, we assessed the effects of events on mood, rather than psychiatric diagnoses, such as major depressive disorder. In addition, because of limited power, we did not examine demographic differences beyond gender. Future investigation of the role of other characteristics, including race, ethnicity, national origin, immigration status, sexual orientation, religion, and political affiliation would be beneficial. Finally, we focused on the US only. Similar dramatic societal events have occurred in other countries, and it is unclear how such exogenous factors affected their physician workforces.

## Conclusion

In this investigation of the contemporary effects of political events on the emotional state of young physicians using long term mood data from the Intern Health Study, we observed a statistically significant reduction in mood for the 2016 presidential election and most political events that followed. These findings suggest that in the current era, macro-level factors such as politics may affect the mood of young doctors, with some events leading to declines in mood that matched the drop in mood seen with the start of internship. These findings signal that politics and medicine may interact in strong ways in the current era of medicine and that we should carefully consider their implications for young physicians and their patients.

**What is already known about this topic**

- Heavy workloads, medical errors, and sleep deprivation are systematic factors that affect the mental health of training physicians
- Physicians’ political beliefs can influence decisions about patient care
- Debates about healthcare and other politicised social issues have become increasingly prominent and polarised in the contemporary era, yet the potential implications for physician mental health are unknown

**What this study adds**

- In this longitudinal cohort study, contemporary political events were associated with a change in mood among young physicians
- The greatest changes in mood were decreases observed in association with the 2016 presidential election and subsequent inauguration, with women experiencing more than twice the mood decline as men following both events
- In contrast to political events, non-political events were not associated with a change in mood

**Patient and public involvement**

No patients were involved in setting the research question or the outcome measures for this study, nor were they involved in developing plans for recruitment, design, or implementation. No patients were asked to advise on interpretation or writing up of results. The results will be disseminated to participants through electronic newsletter, the study website, press release, and social media.

**Figure:**
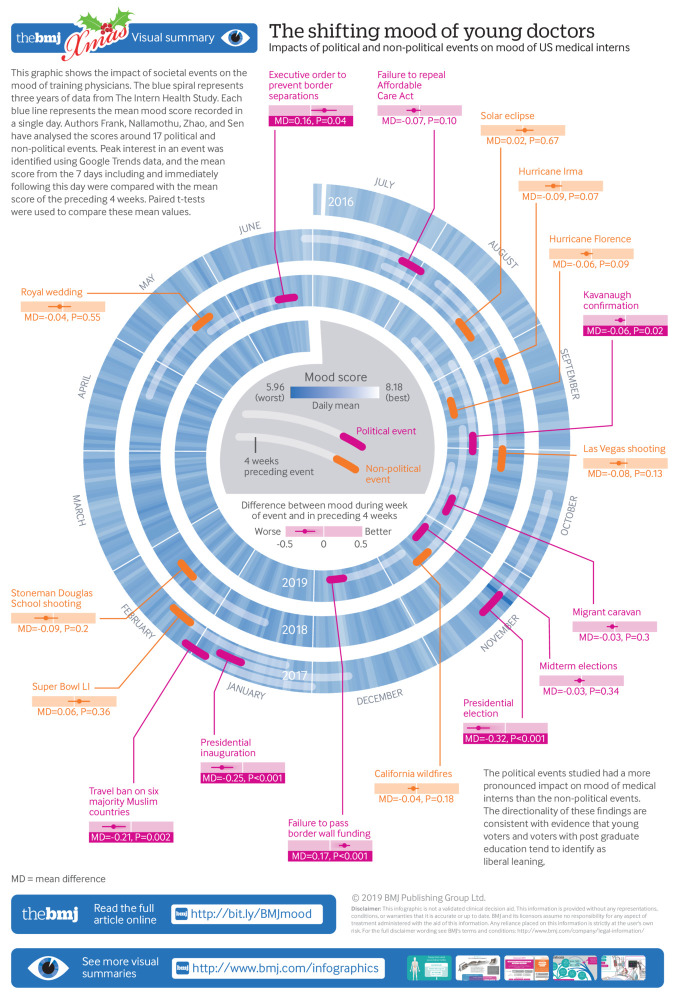


## Web extra

Extra material supplied by authors and BMJ infographic

Supplementary table: Terms used to query Google Trends to determine date of peak search interest for political and non-political events

Infographic: A visual summary of impacts of political and non-political events on mood of US medical interns

## Notes

We thank the physicians who took part in the study. We also thank Dr John Ayanian for his valuable insights.

Contributors: This study was designed by EF and SS. EF managed data collection. ZZ conducted the statistical analyses. EF, BN, and SS were responsible for interpreting the data. EF and SS wrote the initial manuscript draft, and BN provided critical revisions. SS obtained funding. All authors approved the final manuscript and had full access to the data. The corresponding author attests that all listed authors meet authorship criteria and that no others meeting the criteria have been omitted. EF and SS are the guarantors.

Ethical approval: This study was approved by the University of Michigan Medical School Institutional Review Board (HUM00033029).

Data sharing: Dataset available from the corresponding author at srijan@umich.edu.

Transparency: The manuscripts guarantors (EF, SS) affirm that this manuscript is an honest, accurate, and transparent account of the study being reported; that no important aspects of the study have been omitted; and that any discrepancies from the study have been explained.

## References

[1] Mata DA Ramos MA Bansal N Prevalence of depression and depressive symptoms among resident physicians: a systematic review and meta-analysis. JAMA 2015;314:2373-83. 10.1001/jama.2015.15845 26647259 PMC4866499

[2] Dyrbye LN West CP Satele D Burnout among U.S. medical students, residents, and early career physicians relative to the general U.S. population. Acad Med 2014;89:443-51. 10.1097/ACM.0000000000000134 24448053

[3] Buddeberg-Fischer B Stamm M Buddeberg C Klaghofer R [Anxiety and depression in residents - results of a Swiss longitudinal study] [in German]. Z Psychosom Med Psychother 2009;55:37-50. 10.13109/zptm.2009.55.1.37 19353511

[4] Association of American Medical Colleges. Enrollment, graduates, and MD-PhD data. 2018. https://www.aamc.org/data/facts/enrollmentgraduate/.

[5] Bonica A Rosenthal H Rothman DJ The political polarization of physicians in the United States: an analysis of campaign contributions to federal elections, 1991 through 2012. JAMA Intern Med 2014;174:1308-17. 10.1001/jamainternmed.2014.2105 24887456

[6] Henry J Henry J. Kaiser Family Foundation Commonwealth Fund Experiences and attitudes of primary care providers under the first year of ACA coverage expansion: findings from the Kaiser Family Foundation/Commonwealth Fund 2015 National Survey of Primary Care Providers. Issue Brief (Commonw Fund) 2015;17:1-21. 26103743

[7] Frank E Carrera J Dharamsi S Political self-characterization of U.S. medical students. J Gen Intern Med 2007;22:514-7. 10.1007/s11606-007-0108-5 17372802 PMC1829428

[8] Hersh ED Goldenberg MN Democratic and Republican physicians provide different care on politicized health issues. Proc Natl Acad Sci U S A 2016;113:11811-6. 10.1073/pnas.1606609113 27698126 PMC5081578

[9] Sen S Kranzler HR Krystal JH A prospective cohort study investigating factors associated with depression during medical internship. Arch Gen Psychiatry 2010;67:557-65. 10.1001/archgenpsychiatry.2010.41 20368500 PMC4036806

[10] Nuti SV Wayda B Ranasinghe I The use of google trends in health care research: a systematic review. PLoS One 2014;9:e109583. 10.1371/journal.pone.0109583 25337815 PMC4215636

[11] Kalmbach DA Fang Y Arnedt JT Effects of sleep, physical activity, and shift work on daily mood: a prospective mobile monitoring study of medical interns. J Gen Intern Med 2018;33:914-20. 10.1007/s11606-018-4373-2 29542006 PMC5975162

[12] Foreman AC Hall C Bone K Cheng J Kaplin A Just text me: using SMS technology for collaborative patient mood charting. J Particip Med 2011;3:e45.

[13] History.com. 2017 Events. 2017. https://www.history.com/topics/21st-century/2017-events.

[14] History.com. 2018 Events. 2018.https://www.history.com/topics/21st-century/2018-events.

[15] Bellini LM Baime M Shea JA Variation of mood and empathy during internship. JAMA 2002;287:3143-6. 10.1001/jama.287.23.3143 12069680

[16] Pew Research Center. Party Affiliation among voters: 1992-2016. 2016. https://www.people-press.org/2016/09/13/2-party-affiliation-among-voters-1992-2016/.

[17] Federal Election Commission. Federal Elections 2016: Election Results for the US president, the US Senate, and the US House of Representatives. 2017. https://transition.fec.gov/pubrec/fe2016/federalelections2016.pdf.

[18] Neria Y Sullivan GM Understanding the mental health effects of indirect exposure to mass trauma through the media. JAMA 2011;306:1374-5. 10.1001/jama.2011.1358 21903818 PMC3637659

[19] Waismel-Manor I Ifergane G Cohen H When endocrinology and democracy collide: emotions, cortisol and voting at national elections. Eur Neuropsychopharmacol 2011;21:789-95. 10.1016/j.euroneuro.2011.03.003 21482457

[20] Stanton SJ Labar KS Saini EK Kuhn CM Beehner JC Stressful politics: voters’ cortisol responses to the outcome of the 2008 United States Presidential election. Psychoneuroendocrinology 2010;35:768-74. 10.1016/j.psyneuen.2009.10.018 19962831

[21] Trawalter S Chung VS DeSantis AS Simon CD Adam EK Physiological stress responses to the 2008 US presidential election: The role of policy preferences and social dominance orientation. Group Process Intergroup Relat 2012;15:333-45 10.1177/1368430211428163 .

[22] Pitcho-Prelorentzos S Kaniasty K Hamama-Raz Y Factors associated with post-election psychological distress: The case of the 2016 U.S. presidential election. Psychiatry Res 2018;266:1-4. 10.1016/j.psychres.2018.05.008 29787806

[23] Hoyt LT Zeiders KH Chaku N Toomey RB Nair RL Young adults’ psychological and physiological reactions to the 2016 U.S. presidential election. Psychoneuroendocrinology 2018;92:162-9. 10.1016/j.psyneuen.2018.03.011 29606376

[24] Krupenkin M Rothschild D Hill S Yom-Tov E President Trump stress disorder: partisanship, ethnicity, and expressive reporting of mental distress after the 2016 election. SAGE Open 2019;9:2158244019830865 10.1177/2158244019830865 .

[25] Gemmill A Catalano R Casey JA Association of preterm births among US Latina women with the 2016 presidential election. JAMA Netw Open 2019;2:e197084. 10.1001/jamanetworkopen.2019.7084 31322687 PMC6647358

[26] Williams DR Medlock MM Health effects of dramatic societal events—ramifications of the recent presidential election. N Engl J Med 2017;376:2295-9. 10.1056/NEJMms1702111 28591522

[27] Booth W, Adam K. Britain braces for an exodus of EU doctors and nurses feeling hurt by Brexit. *The Washington Post* 2018 https://www.washingtonpost.com/world/europe/britain-braces-for-an-exodus-of-eu-doctors-and-nurses-shaken-by-brexit/2018/03/04/a0e41862-09d0-11e8-998c-96deb18cca19_story.html.

[28] World Medical Association. Declaration of Geneva: The “Modern Hippocratic Oath”. 2019. https://www.wma.net/what-we-do/medical-ethics/declaration-of-geneva/.

[29] Lilienfeld SO Miller JD Lynam DR The Goldwater Rule: Perspectives from, and implications for, psychological science. Perspect Psychol Sci 2018;13:3-27. 10.1177/1745691617727864 29024609

