# Supplementary material for: Political events and mood among young physicians: a prospective cohort study
Source: BMJ. 2019 Dec 9;367:l6322. doi: 10.1136/bmj.l6322 (PMC7190044; doi:10.1136/bmj.l6322)
Supplement: Supplementary file 1 — Supplementary table: Terms used to query Google Trends to determine date of peak search interest for political and non-political events [file frae051959.ww1.pdf]

SUPPLEMENTARY MATERIAL

Supplemental Table 1 Terms used to query Google Trends to determine date of peak search interest for political and non-political events

**Political Events**	**Google Trends Search Terms**
Presidential Election	president + election + trump
Presidential Inauguration	president + inauguration + trump
Muslim Ban	muslim + travel + ban
Failure to Repeal ACA	aca + obamacare + affordable care act + repeal + fail
Executive Order to Prevent Border Separations	border + separation + children
Kavanaugh Confirmation	kavanaugh + confirmation
Migrant Caravan	migrant + caravan
Midterm Elections	midterm + election
Failure to Pass Border Wall Funding	border + wall + budget + fail

**Non-Political Events**	**Google Trends Search Terms**
Super Bowl	super + bowl + 2017
Solar Eclipse	total + solar + eclipse
Hurricane Irma	hurricane + irma
Las Vegas Shooting	las vegas + shooting
Stoneman Douglas Shooting	florida + school + shooting
Royal Wedding	royal + wedding + meghan + harry
Hurricane Florence	hurricane + florence
California Wildfires	california + fire
