# Supplementary material for: Political events and mood among young physicians: a prospective cohort study
Source: BMJ. 2019 Dec 9;367:l6322. doi: 10.1136/bmj.l6322 (PMC7190044; doi:10.1136/bmj.l6322)

thebmj Xmas Visual summary

The shifting mood of young doctors

Impacts of political and non-political events on mood of US medical interns

This graphic shows the impact of societal events on the mood of training physicians. The blue spiral represents three years of data from The Intern Health Study. Each blue line represents the mean mood score recorded in a single day. Authors Frank, Nallamothu, Zhao, and Sen have analysed the scores around 17 political and non-political events. Peak interest in an event was identified using Google Trends data, and the mean score from the 7 days including and immediately following this day were compared with the mean score of the preceding 4 weeks. Paired t-tests were used to compare these mean values.

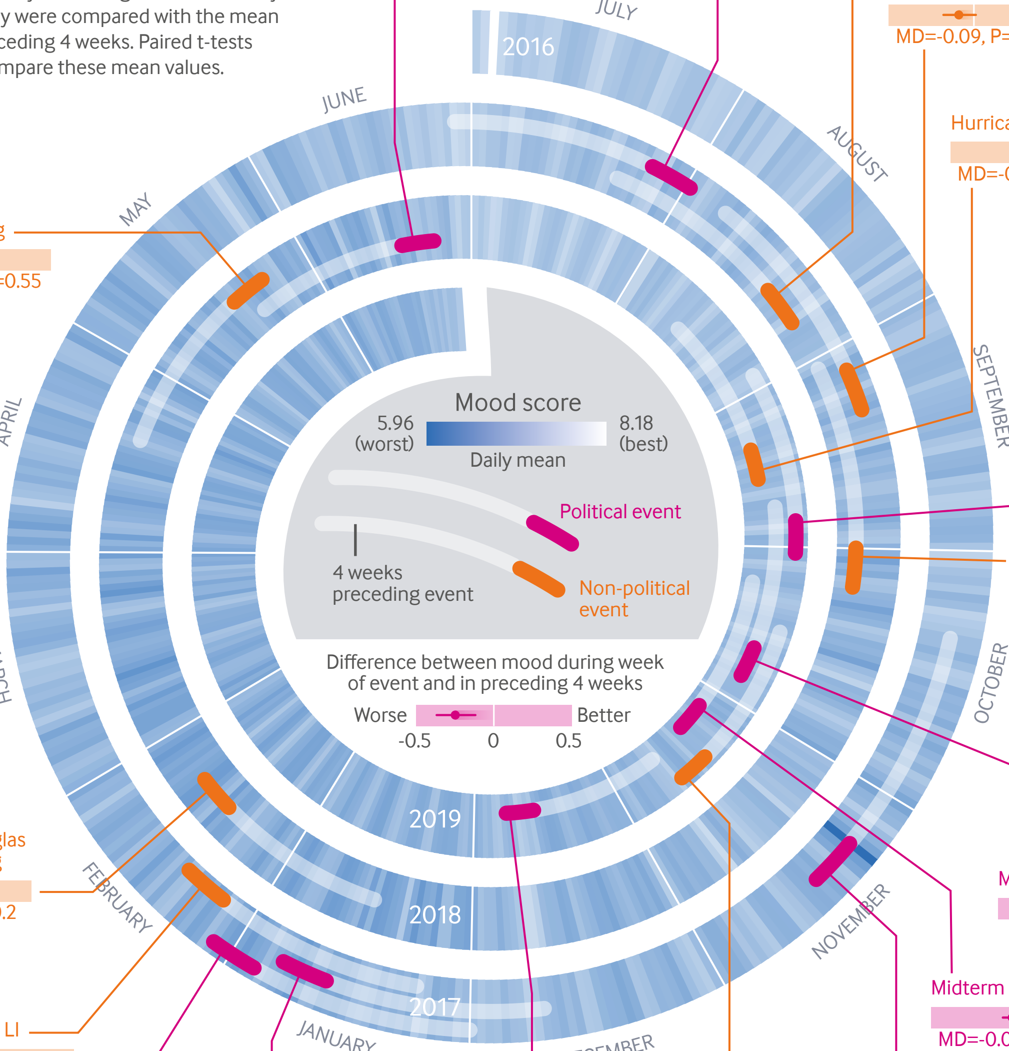

The political events studied had a more pronounced impact on mood of medical interns than the non-political events. The directionality of these findings are consistent with evidence that young voters and voters with post graduate education tend to identify as liberal leaning,

MD = mean difference

thebmj Read the full article online http://bit.ly/BMJmood

© 2019 BMJ Publishing Group Ltd.

Disclaimer: This infographic is not a validated clinical decision aid. This information is provided without any representations, conditions, or warranties that it is accurate or up to date. BMJ and its licensors assume no responsibility for any aspect of treatment administered with the aid of this information. Any reliance placed on this information is strictly at the user's own risk. For the full disclaimer wording see BMJ's terms and conditions: http://www.bmj.com/company/legal-information/

See more visual summaries http://www.bmj.com/infographics

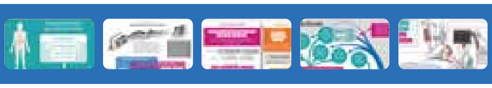

Supplement: Supplementary file 2 — Infographic: A visual summary of impacts of political and non-political events on mood of US medical interns [file frae051959.ww2.pdf]
